# Connecting two proteins using a fusion alpha helix stabilized by a chemical cross linker

**DOI:** 10.1038/ncomms11031

**Published:** 2016-03-16

**Authors:** Woo Hyeon Jeong, Haerim Lee, Dong Hyun Song, Jae-Hoon Eom, Sun Chang Kim, Hee-Seung Lee, Hayyoung Lee, Jie-Oh Lee

**Affiliations:** 1 Department of Chemistry, KAIST, Daejeon 34141, Korea; 2 Department of Biological Sciences, KAIST, Daejeon 34141, Korea; 3 Agency for Defense Development, Daejeon 34186, Korea; 4 Institute of Biotechnology, Chungnam National University, Daejeon 34134, Korea

## Abstract

Building a sophisticated protein nano-assembly requires a method for linking protein components in a predictable and stable structure. Most of the cross linkers available have flexible spacers. Because of this, the linked hybrids have significant structural flexibility and the relative structure between their two components is largely unpredictable. Here we describe a method of connecting two proteins via a ‘fusion α helix' formed by joining two pre-existing helices into a single extended helix. Because simple ligation of two helices does not guarantee the formation of a continuous helix, we used EY-CBS, a synthetic cross linker that has been shown to react selectively with cysteines in α-helices, to stabilize the connecting helix. Formation and stabilization of the fusion helix was confirmed by determining the crystal structures of the fusion proteins with and without bound EY-CBS. Our method should be widely applicable for linking protein building blocks to generate predictable structures.

## Abstract (web summary)

Linking protein components in a controlled manner is crucial for assembling protein nanostructures with pre-determined architecture. Here, the authors use a chemical cross-linker to fuse the terminal helices of two proteins into a single one, forcing the protein domains in a specific orientation.

## Graphical abstract

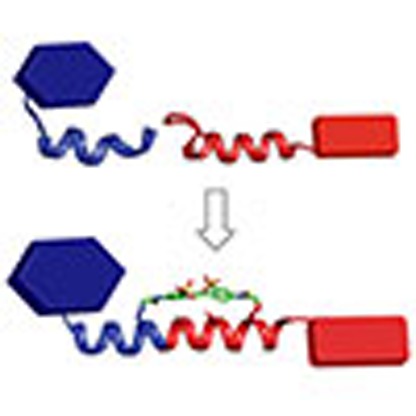

Linking two protein components to form a predictable and rigid structure is a prerequisite for generating complex protein assemblies in a pre-designed fashion^1^. Most of the chemical cross linkers available have long and flexible spacers to help them approach the reactive side chains of the target proteins. Because of this, the resulting hybrids have significant structural flexibility and the relative orientation and distance between their two components is largely unpredictable. This is the case even when the chemical cross linkers themselves have rigid structures since they are attached to flexible side chains such as cysteines or lysines. Recently several new methods have been proposed to assemble proteins in a pre-designed fashion.

Radford *et al*.^2^,^3^,^4^ have shown that two proteins can be linked in a rigid and predictable way using a metal coordination method. Since the bound metal ion has additional coordination sites that are available for binding of another protein molecule, it can induce homodimerization of the protein. Because the metal coordination requires precise positioning of the coordinating amino acid side chains, the resulting protein dimer will have a predictable structure. The authors used a similar metal coordination method to produce a variety of protein assemblies^5^,^6^,^7^,^8^,^9^,^10^,^11^,^12^. King *et al*.^13^ reported a computational method for designing cage-like structures by connecting two protein components. One of them was chosen from among natural proteins that can form stable homodimers and the other from among proteins that can form stable homotrimers. By optimizing the interface between these two protein components in the fusion proteins using computer calculations, they were able to produce several fusion proteins that could assemble into large cage-like structures.

Short alpha-helical linkers joining two protein components have been used to build large and highly symmetric assemblies with rigid and predictable structures. Because an α-helix is under strict structural restraint, the structure of the fusion protein ought to be predictable so long as a connecting helix is formed. Diverse architectures are possible, depending on the specific geometric arrangement between the protein components^14^. Using this method, Lai *et al*.^1^,^15^,^16^ connected M1 matrix protein, which can form homodimers, and BPO protein, which can form stable homotrimers, with a short linker. By testing various linker sequences they were able to find one that could form an α-helix and supported the formation of cage-like structures. Recently, they applied a similar method to generate large and porous cube-like closed structures with high accuracy^17^. Rigidity of the helical linker is essential for the success of the design because apparently minor deviation of the structure from the intended ideal helix can lead to failure of protein assembly^18^.

Here we propose a novel method for connecting the pre-existing α-helices of two proteins into a single extended helix using a chemical cross linker. To test our method, we chose as a model system an ankyrin family protein containing a C-terminal helix and a protein A fragment containing an N-terminal helix. We fused the two proteins by connecting the two terminal α-helices into a single continuous helix. In general, simple genetic ligation of the helices in the proteins does not lead to fusion of the helices. Therefore, we used a synthetic chemical that selectively reacts with α helices and stabilizes them. EY-CBS (3,3′-ethyne-1,2-diylbis-{6-[(chloroacetyl)amino]benzenesulfonic acid}) is a chemical cross linker that can react simultaneously with two cysteines in the i and i+11 positions of the α-helix^19^. To test whether EY-CBS detects the formation of a helix, we chose two amino acid residues separated by 11 amino acids in the proposed fusion helix and mutated them to cysteines. The successful fusion helices showed high reactivity with EY-CBS, and cross linking with EY-CBS forced the structure of the fusion α-helix to resemble that of an ideal helix. Our method does not require metal ions in the buffer, nor complicated computer simulation in the design process. It can be used to assemble protein components into asymmetrical and non-repeating structures.

## Results

### Design of the fusion helices

**Figure 1:**
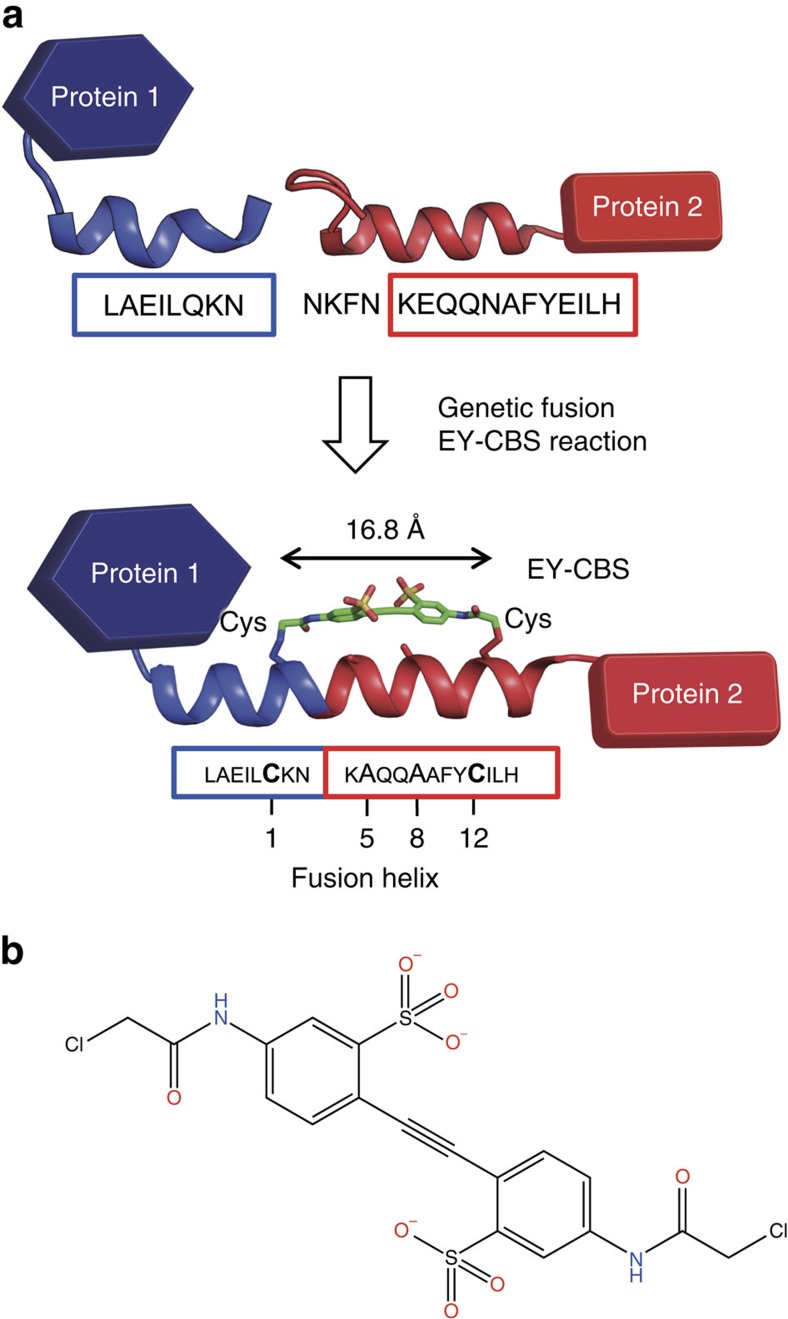
Design of the fusion proteins. (**a**) The C-terminal helix of protein 1 and the N-terminal helix of protein 2 were genetically connected. Two amino acids separated by 11 amino acids were mutated to cysteines for reaction with EY-CBS. (**b**) Chemical structure of EY-CBS. EY-CBS has a rigid chemical structure with two α-chlorocarbonyl groups reactive with the thiol groups of cysteines.

We chose an artificial ankyrin protein, named ‘mbp3-16', that has been engineered to bind maltose-binding protein (MBP)^20^, and the B4 domain of protein A as a model system to test our fusion helix method. We chose these proteins because both of them are composed entirely of α-helices and lack cysteine residues. In addition to this, they have been proven to be useful as building blocks of artificial protein assemblies because they can be engineered to bind other proteins^21^,^22^. We produced 17 fusion proteins by connecting the C-terminal helix of the ankyrin domain and the N-terminal helix of the protein A domain (Fig. 1 and Table 1). While designing connections between the two helices, obvious steric collision was avoided by using a molecular graphics program. To generate the EY-CBS reaction site, two amino acid residues separated by 11 amino acids, one from the ankyrin domain and the other from the protein A domain, were chosen and mutated to cysteines. Also, two additional amino acids, in positions i+4 and i+7, were mutated to alanines because long side chains may lead to steric collision with the EY-CBS molecules^19^. Using a similar method, we designed four more fusion proteins with the protein A domain and the calmodulin N-terminal domain^23^. We intentionally positioned positively charged residues such as lysines and arginines in some of the fusion proteins to test whether the negatively charged EY-CBS had higher reactivity with positively charged targets (Fig. 1b). However, we found no obvious correlation between the location of the positively charged residues and the reactivity of the protein towards EY-CBS, and we did not artificially include positively charged amino acids in the later design. When necessary, we inserted alanine residues between the two proteins to control the relative orientation of the two protein components (Table 1). Note that insertion of one amino acid residue in an α-helix will rotate the helix by ∼100 degrees. Therefore, one can control the relative orientation of the connected proteins by inserting precise number of amino acids into the fusion helix. Amino acid residues that may have roles in stabilizing the native helices were not changed.

### Reaction with EY-CBS

**Figure 2:**
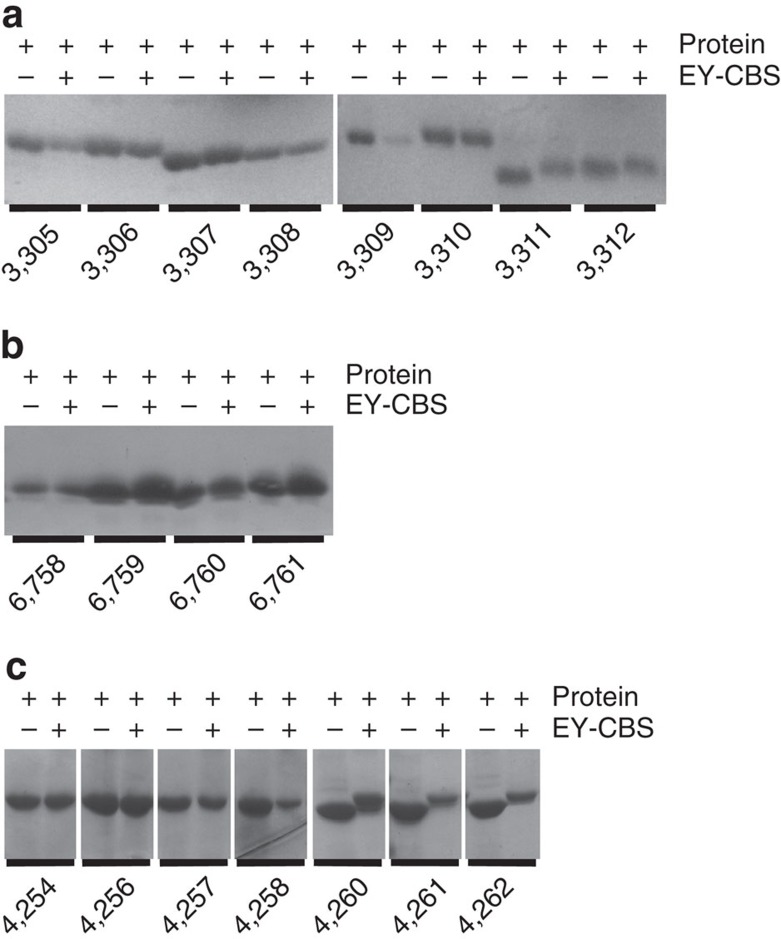
Reactivity of the designed fusion proteins. The protein A-ankyrin (**a**, **c**) and the protein A-caldmodulin (**b**) fusion proteins were treated with 1mM EY-CBS. Constructs 3,311, 3,312, 4,260, 4,261 and 4,262 are substantially upshifted after the reaction.

**Figure 3:**
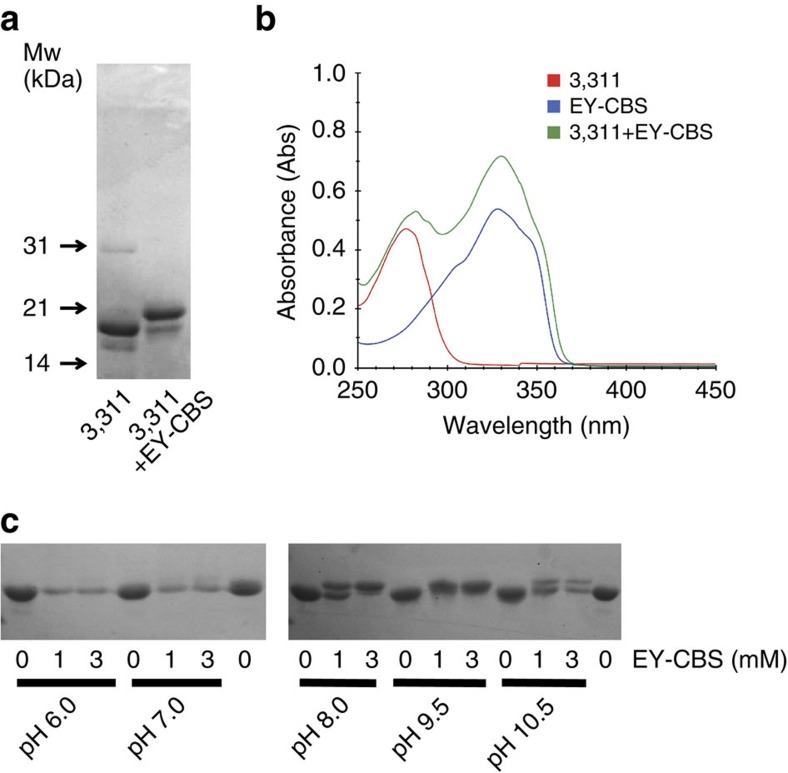
Reactivity of the 3,311 fusion protein toward EY-CBS. (**a**) SDS–PAGE analysis of the 3,311 protein. Nearly 100% of the 3,311 protein is upshifted by reaction with EY-CBS. (**b**) Absorption spectrum of the 3,311 protein reacted with EY-CBS. Excess EY-CBS after reaction was removed by gel filtration chromatography. (**c**) pH dependence of the EY-CBS reaction. The 3,311 protein was reacted with EY-CBS at different pH and analysed by SDS–PAGE.

The fusion proteins produced were purified and incubated with 1 mM EY-CBS (Fig. 2 and Supplementary Figs 1–3). Five of the 21 fusion proteins shifted substantially in the SDS–PAGE analysis. The molecular weight of EY-CBS is 521 daltons and if the two cysteines in the fusion helix react with EY-CBS, the shift in molecular weight should be by 521 daltons. In fact the change of molecular weight estimated from the shift of the protein bands was more than ∼2 kDa (Fig. 3a). Therefore, we assumed the band shift was not only due to the additional molecular weight of the bound EY-CBS but to a change in the local structure of the fusion helix induced by circularization of the two cysteine side chains by EY-CBS. To analyse the situation further, we chose the 3,311 fusion protein because it had the highest reaction efficiency with EY-CBS (Fig. 3 and Supplementary Figs 4–6). To confirm that the 3,311 fusion indeed reacted with EY-CBS, the absorption spectrum of the reacted protein was measured after removing unreacted EY-CBS by gel filtration chromatography. The isolated EY-CBS molecule has an absorption peak at 340 nm due to the conjugated phenyl rings. As expected, the reacted fusion protein had a strong absorption peak at 340 nm, demonstrating that EY-CBS was covalently linked to the protein (Fig. 3b). The ratio of the absorption peaks at 280 and 340 nm suggests that one EY-CBS molecule is bound per protein molecule.

To maximize the reaction efficiency of EY-CBS, we measured the reactivity of the 3,311 protein as a function of pH, anticipating that the deprotonated thiol groups of the cysteines would have higher reactivity at higher pH. As expected, the reaction was strongly dependent on pH and proceeded to near completion at pH 8 (Fig. 3c). At pH >9, the protein band and reaction efficiency declined presumably due to denaturation and precipitation of the fusion protein.

### Crystal structure of the 3,311 fusion protein

**Figure 4:**
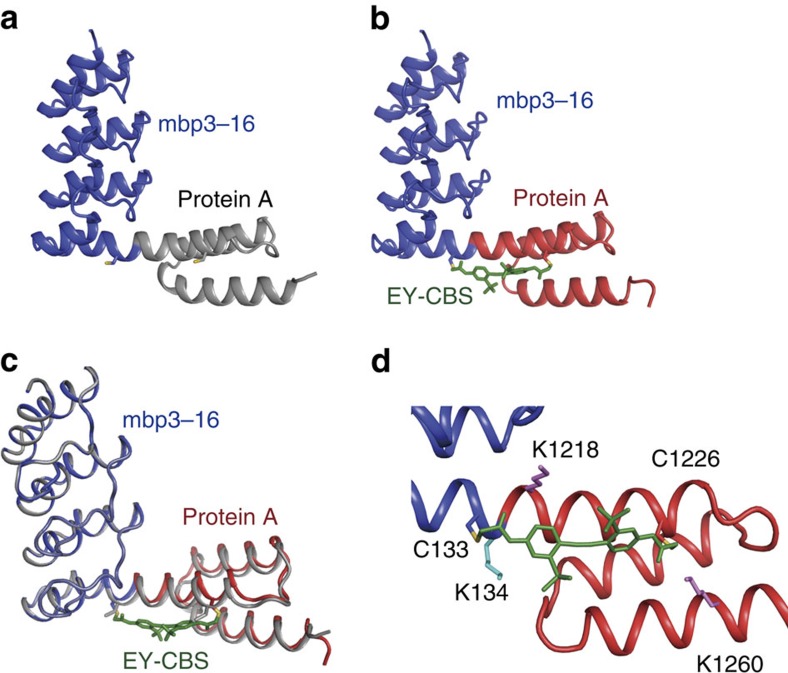
Crystal structure of the 3,311 fusion protein after reaction with EY-CBS. (**a**) The intended structure of the 3,311 fusion protein. The ankyrin domain of mbp3-16 and the protein A domain are connected by an α-helix. (**b**) Crystal structure of 3,311 EY-CBS. EY-CBS is attached to the fusion helix and shown in green. For clarity the structure of the MBP bound to the ankyrin domain is not shown. (**c**) Superimposition of the intended and crystal structures of the 3,311 fusion. The intended and crystal structures are coloured in grey and blue/red, respectively. (**d**) Close-up view of the fusion helix. Positively charged amino acid side chains that interact with the sulfate groups of EY-CBS are shown.

**Figure 5:**
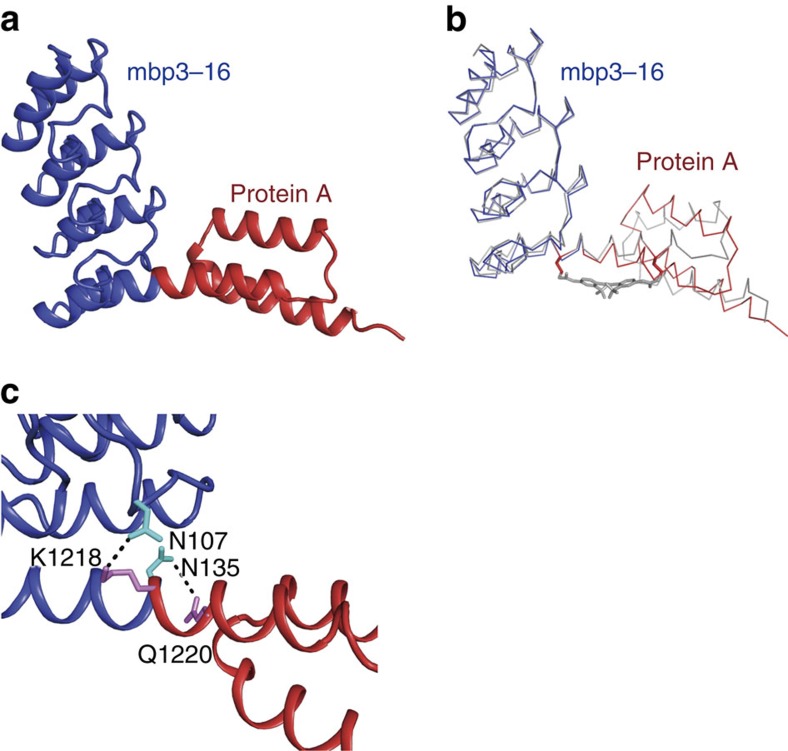
Crystal structure of the 3,311 fusion protein not reacted with EY-CBS. (**a**) Crystal structure of the fusion protein. The fusion helix is bent by ∼20 degrees. (**b**) Superposition of the EY-CBS-bound and -unbound structures of 3,311 fusion protein. The 3,311 EY-CBS and 3,311 structures are coloured in grey and blue/red, respectively. (**c**) Close-up view of the fusion helix. Amino acid residues that may stabilize the fusion helix are labeled.

To verify fusion of the helices, we crystallized the 3,311 fusion protein before and after reaction with EY-CBS and determined the structures of the two products (Figs 4 and 5). Since the ankyrin domain, mbp3–16, had been engineered to bind MBP, we were able to use MBP to facilitate crystallization^20^. In the crystal structure, one MBP molecule interacted exclusively with the mbp3–16 component of the hybrid protein and there was no interaction with either protein A or the fusion helix. The electron density map calculated using the refined protein structure showed a clear density corresponding to the EY-CBS, and one EY-CBS molecule could be modelled into the density (Supplementary Fig. 7). Both reactive ends of the EY-CBS are covalently linked to the cysteines in the fusion helix. In the crystal structure, the fusion helix adopts a nearly ideal α-helical structure and matches closely to the intended structure (Fig. 4c). The Cα atoms of the cysteines are separated by 16.8 angstrom and the mutated alanines are located under the phenyl rings of EY-CBS, as designed. The structures of the ankyrin and protein A domains are easily superimposable on those of the isolated proteins, which demonstrates that the helix fusion has no impact on the structure of the protein other than near the fusion site (Fig. 4b and c). The sulfate moieties of the EY-CBS groups form nonspecific ionic interactions with three lysine groups of the protein A and ankyrin domains (Fig. 4d). Other than that, EY-CBS does not have any noticeable interaction with the protein.

We determined the crystal structure of the unreacted 3,311 fusion protein at 2.8 angstrom resolution to understand why it reacts strongly with EY-CBS (Fig. 5). MBP was not included in the crystallized protein solution. The crystal structure showed that the protein A and ankyrin domains were connected by a slightly bent helix (Fig. 5a). When the structures of the fusion helix with and without the bound EY-CBS were superimposed, the fusion helix was seen to be bent by ∼20 degrees with respect to that of the EY-CBS-bound helix (Fig. 5b). The asymmetric unit of the crystal contains 12 fusion proteins that can be divided into two groups, each group containing 6 proteins (Supplementary Fig. 8). The proteins belonging to the same group are in a similar crystal environment and, as expected, have superimposable structures. However, the proteins belonging to different groups are in completely different crystal environments. Despite this, the proteins in the different groups have almost identical structures, with a Cα root mean square difference value of 0.32 angstrom. Furthermore, the simulated annealing omit map calculated using the refined protein structure showed a clear density near the fusion helix (Supplementary Fig. 9). Because of these, we conclude that formation of the fusion helix is unlikely to be a crystallization artifact.

Crystal structure of the unreacted 3,311 clearly explains why the 3,311 fusion protein is strongly reactive with EY-CBS: the fusion helix is already present before EY-CBS is bound; after the covalent reaction with EY-CBS the fusion helix resembles more closely the ideal helix because the distance between the two cysteines is forced to be 16.8 angstrom. In the crystal structure, Asn107 and Lys1218 are separated by 2.9 angstrom and are hydrogen bonded. In addition, Asn135 is connected to Gln1220 via a hydrogen bond and contributes to stability of the fusion helix (Fig. 5c). However, it is not clear whether these relatively weak interactions are enough to stabilize the helical connection between the two protein components. More sophisticated calculations are required to analyse this stabilization.

### Crystal structure of a fusion protein that failed the EY-CBS test

Of the 21 fusion proteins, 16 failed to display any significant reactivity towards EY-CBS (Fig. 2). We were unable to crystallize any of these proteins except 6,761, presumably due to the structural instability of the interface between the fused protein components.

**Figure 6:**
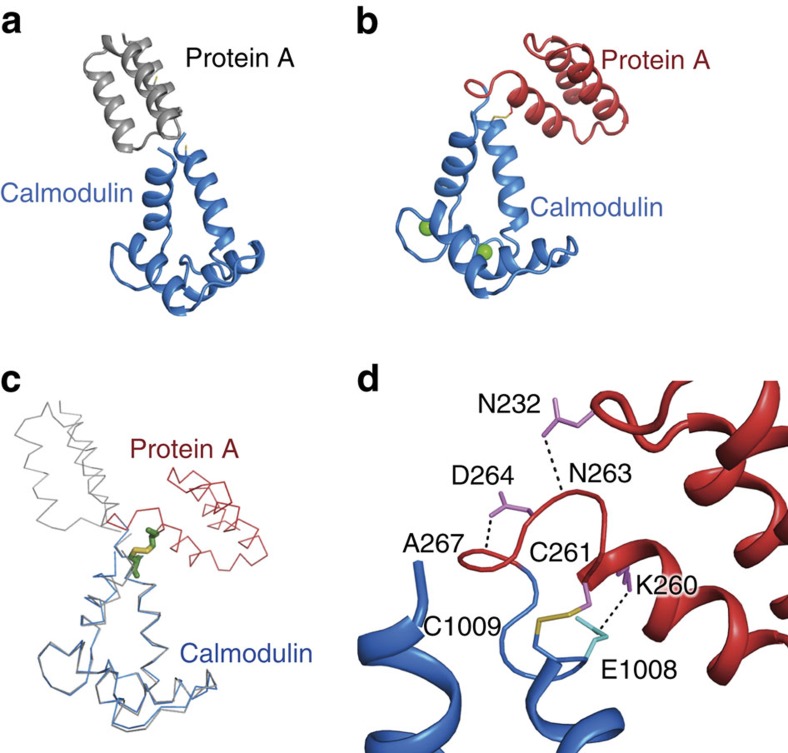
Crystal structure of the 6,761 fusion protein containing the protein A domain and the calmodulin domain. (**a**) Intended structure of the 6,761 fusion protein. The two protein domains are connected by a continuous α-helix. (**b**) Crystal structure of the 6,761 fusion protein. An unexpected disulfide bridge that connects the mutated cysteines is shown. (**c**) Superposition of the intended and crystal structures of the 6,761 fusion protein. The intended structure is coloured in grey and the crystal structure is coloured in blue and red. (**d**) Close-up view of the fusion site. The cysteine 261 of the protein A domain and cysteine 1,009 of the calmodulin domain form a disulfide bridge. Amino acid residues that stabilize the interface of the two helices are drawn and labeled.

The crystallized 6,761 fusion protein is composed of an N-terminal protein A and a C-terminal calmodulin domain (Supplementary Fig. 10). Calmodulin is a calcium sensor composed of two homologous calcium-binding domains. Each calmodulin domain contains two EF hand motifs that bind calcium ions^24^. The crystal structure of the 6,761 fusion protein was determined at 2.7 angstrom resolution (Fig. 6). The structures of the two domains of the 6,761 fusion protein were easily superimposable with those of the separate protein A and calmodulin domains, and there were no noticeable structural changes except around the fusion site. In the crystal structure, it could be seen that the two helices designed to fuse had not formed a continuous helix, and the cysteines introduced to bind EY-CBS were close to one another and formed an unexpected disulfide bridge (Fig. 6c). The last and the first turns of the helices of the protein A and calmodulin domains, respectively, formed irregular loops and made sharp ∼120 degree turns. The crystal structure of the connecting loop and the disulfide bridge formed a distinct electron density demonstrating that the fusion site was structurally stable (Supplementary Fig. 11). It appears that the 6,761 fusion protein can be crystallized because the interface between the two protein components of the fusion protein is stabilized by the opportunistic disulfide bridge. Several strong interactions can be seen at the fusion site and these account for why the disulfide bridge is formed (Fig. 6d). Lys260 of the protein A domain and Glu1008 of the calmodulin domain are separated by only 3.8 angstrom and form a strong ionic interaction. In addition, hydrogen bonding of the backbone nitrogen of Ala267 and the carboxylic oxygen of Asp264 should also contribute to the stabilization of the disulfide bridge. Furthermore, the side chain of Asn232 and the backbone carbonyl oxygen of Asn263 are separated by 2.9 angstrom and form a strong hydrogen bond.

We conclude that that simple-minded ligation of two helices does not guarantee the formation of a single extended helix, and that high reactivity of the fusion helix with EY-CBS is a reliable indicator of helix formation.

### Insertion of a binding adaptor protein into an internal loop

To confirm the validity of our EY-CBS method, we designed several fusion proteins containing the protein A domain inserted into loops preceding internal rather than terminal α-helices of T4 lysozyme. To identify the best insertion site, we tested four loop regions and inserted an artificial protein A analogue, Ztaq (Supplementary Fig. 12)^25^. Three of the four fusion proteins were produced as efficiently as wild-type lysozyme when expressed in *Escherichia coli*. The exception was the 8,133 fusion. All three fusion proteins eluted as monomers in size-exclusion chromatography and were resistant to limited subtilisin digestion, confirming their structural integrity. Of the three possible insertion sites, we chose the loop containing residue 37 and designed three helix-fusion proteins, 8,155, 8,157 and 8,158 containing the EY-CBS-binding sites (Supplementary Figs 13–15). Fusion protein 8,157 has two more amino acids in the fusion helix than protein 8,155 and therefore its protein A domain should be rotated by ∼200 degrees and translated by ∼7 angstroms. Fusion proteins 8,157 and 8,158 have the same EY-CBS sites except that the positions of the reactive cysteines are shifted by four amino acids.

**Figure 7:**
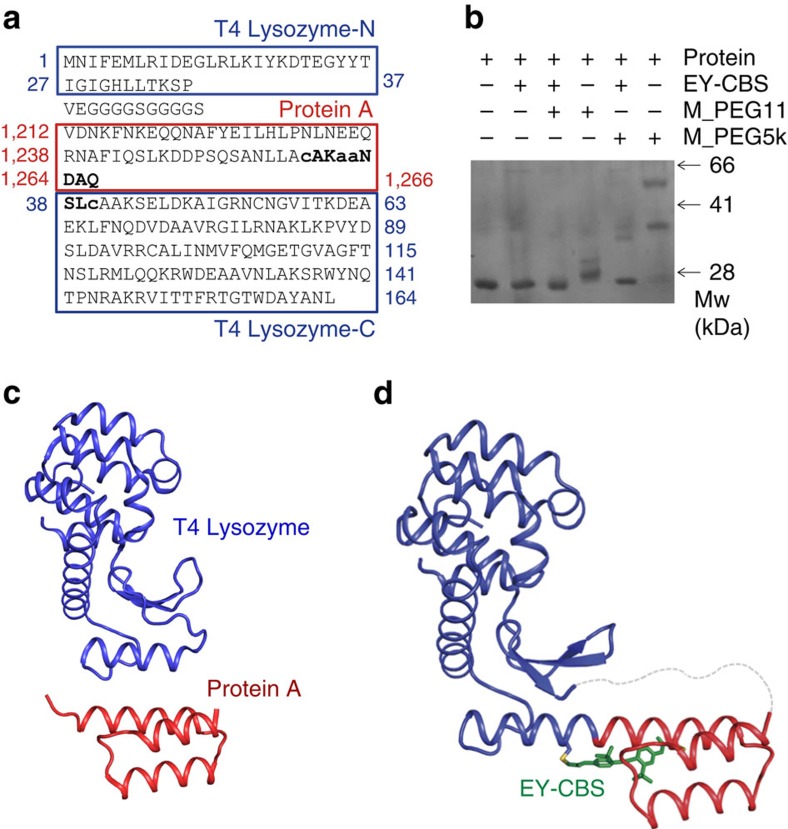
Insertion of the protein A domain into an internal loop of T4 lysozyme. (**a**) The amino acid sequence of fusion protein 8,155. (**b**) Reactivity of fusion protein 8,155 with EY-CBS. After one hour of the reaction, PEG-Maleimide reagent Maleimide-PEG_11_-Biotin (M_PEG11) or PEG-Maleimide 5000 (M_PEG5k) was added where appropriate. (**c**) Previously reported crystal structures of T4 lysozyme and protein A. (**d**) Crystal structure of fusion protein 8,155. The T4 lysozyme and the protein A parts of the fusion protein are coloured in blue and red, respectively. The ‘GGGGS' linker and the N-terminal seven amino acids of protein A that are disordered in the crystal structure are drawn as a broken line.

All three helix-fusion proteins were produced in *E. coli* and purified to homogeneity. Unexpectedly, reaction with EY-CBS did not result in clearly visible upshifts of the protein bands on SDS–PAGE presumably because size of the fusion proteins are too big and resolution of the SDS–PAGE analysis is not good enough to detect small changes in the structure (Fig. 7b and Supplementary Figs 16 and 17). However, we believe that all three proteins had reacted with EY-CBS with high efficiency because their cysteines became resistant to two maleimide-containing reagents, Maleimide-PEG_11_-Biotin and PEG-Maleimide 5000. These reagents are highly reactive with the free thiol groups of cysteines, and their reactivity is easier to detect after SDS–PAGE because they have high-molecular weights, 1.1 and 5 kDa, respectively. As shown in Fig. 7b and Supplementary Figs 16 and 17, the fusion proteins were resistant to the PEG-maleimide reagents after EY-CBS treatment, presumably because they had already formed covalent bonds with EY-CBS, whereas the SDS–PAGE bands formed by the same fusion proteins not reacted with EY-CBS were clearly shifted upwards, showing that their cysteines were free to react with the PEG-maleimide reagents.

To confirm fusion of the α-helices connecting protein A and lysozyme, we crystallized 8,155 after reaction with EY-CBS and determined its crystal structure. The purified and reacted 8,157 and 8,158 fusions were also crystallized, but we have not tried to optimize the crystallization conditions nor determined their structures. The 8,155 crystals diffracted X-rays towards 2.7 angstrom resolution. In the crystal structure, one EY-CBS molecule is covalently connected to the two cysteines in the fusion helix as expected (Supplementary Fig. 18). The distance between the Cα atoms of the reacted cysteines is 16.7 angstrom, which is shorter by only 0.1 angstrom than that of 3,311 treated with EY-CBS. The fusion helix thus adopts a nearly ideal α-helical structure and closely matches the intended structure. The lysozyme and protein A parts of the structure can be superimposed with the structures of the individual proteins, demonstrating that fusion of the two helices had little impact on the overall structure of the individual protein components (Supplementary Fig. 19).

We chose the protein A domain as the insertion partner because it can be mutated to bind a variety of target proteins, as shown previously^26^. Because of this, it can be used as a universal adaptor protein mediating dimerization of pairs of target proteins. Provided we identify a suitable connecting helix, we can use the same helix to connect all other mutant protein A's for the following reasons. First, all the mutant protein A proteins adopt an essentially identical conformation, as shown by many crystal and NMR structures. Second, the C-terminal helix where our EY-CBS site is located is not changed in the mutant proteins because the mutations are limited to the first two α-helices. Among the known mutants, the Ztaq and anti-Ztaq proteins were selected for our study because they can form stable heterodimers^25^. To confirm that the mutations in the Ztaq and anti-Ztaq proteins do not affect the EY-CBS reaction, we replaced the protein A regions of fusions 8,155, 8,157 and 8,158 with the Ztaq or anti-Ztaq domain. The substituted fusion proteins retained similar reactivity with EY-CBS, as shown in Supplementary Fig. 16. Because the structure of the Ztaq-anti-Ztaq heterodimer was already known^25^, we could predict the structures of the homo- and heterocomplexes 8,155–8,155, 8,157–8,157 and 8,155–8,157 with considerable confidence (Supplementary Fig. 20).

Thus, we have shown that universal adaptor proteins such as protein A or a coiled-coil domain can be inserted into a target protein to generate a predictable homo- or heterodimeric structure using our EY-CBS method.

## Discussion

In this research, we have developed a novel method for connecting two proteins using a rigid fusion helix that is stabilized by a chemical cross linker. Using this method, we successfully fused the C-terminal helix of an ankyrin family protein to the N-terminal helix of a protein A fragment. We also succeeded inserting a protein A domain inside T4 lysozyme with predictable structure. Subsequent reaction with EY-CBS stabilized the structure of the fusion helix to that of an ideal helix. Our method, in principle, can be applied to majority of proteins as long as they contain at least one helix in their structure. In our method, EY-CBS reacts with thiol groups of cysteines. Therefore, proteins with natural cysteines may have unwanted reactions with EY-CBS. In such cases all the cysteines exposed to the surface need to be mutated before applying the method. Fortunately, most exposed cysteines found in proteins are not important functionally or structurally and can be converted into other amino acids without deleterious effects. In the PDB database of more than 100,000 protein structures, the majority have at least one α-helix and could therefore be used as building blocks for assembly of complex nanostructures by the helix-fusion method. Furthermore, application area of the helix-fusion method can be expanded by combining it with other methods like the metal chelation or the interface design method.

Several chemicals besides EY-CBS have been reported to stabilize the α-helical content of peptides^27^. Here are some notable examples of these helix stabilizers that can be applied to full size proteins. Two bisarylmethylene bromides named Bph and Bpy can selectively cross-link cysteines at the i and i+7 positions in the α-helices^28^; the cross-linked peptides show increased helicity and cell permeability. Also spermine contains positively charged amines at 4.6 angstrom and 6.0 angstrom spacings, and has been shown to stabilize a peptide with four negatively charged glutamate or aspartate residues separated by 3–4 residues^29^. The stabilizing effect is highly dependent on pH, suggesting that ionic interactions play the major role in the helix stabilization. Similarly a diguanidium-containing compound has been shown to enhance the helical content of a peptide containing aspartate groups in the i and i+3 positions^30^. These compounds may be able to replace EY-CBS in the helix-fusion method. Unlike EY-CBS, some of these helix stabilizing compounds do not use cysteines for cross linking. Therefore, they may be useful for connecting proteins with reactive cysteines.

Crystallization chaperones are proteins that can be easily produced and crystallized^31^,^32^. Fusing them to a target protein has proven successful in improving the crystallization property of the target protein. For example, T4 lysozyme has been inserted into a flexible loop of the β2-adrenergic receptor^33^,^34^. The authors generated a panel of fusion proteins and chose one where the linker between lysozyme and the receptor was rigid enough for crystallization. A similar method using cytochrome cb562 instead of T4 lysozyme has been essential for crystallizing several G-protein-coupled receptor receptors and determining their structures^35^,^36^,^37^,^38^. The success of this method relies on structural rigidity of the fusion site, because proteins with flexible structures cannot readily be crystallized. Since it is generally impossible to predict the structure of fusion sites, a large number of fusion proteins have to be screened and the linker sequence optimized for crystallization. Our method can be used to test whether a fusion protein has a rigid fusion site before extensive and time-consuming crystallization trials. For example, one can insert a helical chaperone protein like cytochrome cb562 into a loop preceding or following an α-helix in the target protein and connect the α-helices of the target and the chaperone protein using EY-CBS as shown in the crystal structure of 8,155 (Fig. 7). The resulting fusion protein will have a rigid helical connection and there will be more chance of successful crystallization.

In this study, we showed that reaction with EY-CBS could force the structure of a fusion helix into that of an ideal helix. The crystal structure demonstrated that the fusion helix was slightly bent at the fusion site before reaction with EY-CBS and was converted to a near-ideal helix after reaction with EY-CBS. For applications that involve crystallization of the fusion protein, the rigidity of the fusion protein is critical and reaction with EY-CBS would be desirable. However, in other applications small deviations from the ideal helical structure and some structural flexibility may be allowed. In such cases the EY-CBS reaction could be useful as a probe to test whether the two helices are indeed fused and a hybrid helix has been formed.

In conclusion, we have developed a new method that can convert two pre-existing α-helices into one long extended helix. This method can be used to link proteins to form a desired structure and may be useful in constructing complex protein nanoassemblies for a variety of purposes.

## Methods

### Design of fusion proteins

The fusion helices between pairs of proteins were designed using the molecular graphics program, COOT^39^, assuming that their structures would not be significantly perturbed by the fusion. The coordinate files of the individual protein components were obtained from the PDB database^20^,^23^,^40^,^41^. The fusion helix was modelled by aligning the helices from the individual proteins with that of an ideal α-helix. Possible clashes between the two proteins were visually checked and avoided by addition or deletion of amino acid residues. Amino acid positions that were open to the solvent for easy approach of EY-CBS were chosen visually and mutated to cysteines for the EY-CBS reaction. Computer programs predicting protein structure were not used in our modelling.

### Protein expression and purification

The genes of the fusion proteins were cloned into pET28a vector and over-expressed using *E. coli* strain BL21(DE3). Protein production was induced by adding 0.5 mM IPTG when OD 600 of the culture reached 0.7 and incubating the cells for an additional 20 h at 25 °C.

For proteins 3,305–3,315, glutathione-*S*-transferase was used as the purification tag. The cells were harvested by centrifugation, resuspended in lysis buffer containing 50 mM Tris pH 8.0, 200 mM NaCl and 10 mM β-mercaptoethanol and homogenized using a microfluidizer, model M-110L (Microfluidics). The supernatant after centrifugation was injected into a Glutathione-Sepharose (GE Healthcare) column equilibrated with a buffer containing 20 mM Tris pH 8.0, 200 mM NaCl and 1 mM dithiothreitol (DTT). The glutathione-*S*-transferase-tagged protein was eluted in a buffer containing 50 mM Tris pH 8.0, 100 mM NaCl, 10 mM glutathione and 1 mM DTT. The protein was cleaved overnight by thrombin to remove the purification tag and purified by a Q-Sepharose (GE Healthcare) anion exchange column equilibrated with a buffer containing 20 mM Tris 8.0 and 1 mM DTT. The bound protein was eluted by linear gradient of 1 M NaCl. The fractions containing the target protein were pooled and purified using a Supderdex 200 (GE Healthcare) gel filtration column equilibrated with a buffer containing 20 mM Tris pH 8.0, 200 mM NaCl and 1 mM TCEP.

For proteins 4,254–4,262, chitin-binding domain was used as the purification tag^42^. The harvested cells were homogenized in lysis buffer containing 50 mM Tris pH 8.0, 200 mM NaCl and 1 mM DTT. The supernatant after centrifugation was loaded onto a chitin affinity column (New England Biolab) equilibrated with 20 mM Tris 8.0, 200 mM NaCl and 1 mM DTT. The protein was eluted by thrombin cleavage and purified by a Q-Sepharose anion exchange column equilibrated with a buffer containing 20 mM Tris 8.0 and 1 mM DTT. The bound protein was eluted by linear gradient of 1 M NaCl. The fractions containing the target protein were pooled and purified using a Supderdex 200 gel filtration column equilibrated with a buffer containing 20 mM Tris pH 8.0, 200 mM NaCl and 1 mM TCEP.

Hexa-histidine was used as the purification tag for constructs 6,758–6,761. The harvested cells were homogenized in lysis buffer containing 50 mM Tris pH 8.0, 200 mM NaCl and 1 mM DTT. The supernatant was loaded onto a Ni-NTA affinity column equilibrated with 20 mM Tris 8.0, 200 mM NaCl and 1 mM CaCl_2_. One millimolar DTT was added to the protein immediately after elution using 500 mM imidazole. The protein was cleaved overnight by thrombin to remove the purification tag. The cleaved protein was purified by Q-Sepharose anion exchange chromatography equilibrated with a buffer containing 20 mM Tris 8.0, 1 mM CaCl_2_ and 1 mM DTT. The bound protein was eluted by linear gradient of 1 M NaCl. The fractions containing the target protein were pooled and purified using a Supderdex 200 gel filtration column equilibrated with a buffer containing 20 mM Tris pH 8.0, 200 mM NaCl, 1 mM CaCl_2_ and 1 mM TCEP. The purified proteins were concentrated with an ultrafiltration kit (Amicon) and used for the EY-CBS reaction.

For proteins 8,132–8,136 and 8,155–8,158, hexa-histidine was used as the purification tag. The harvested cells were homogenized in lysis buffer containing 20 mM Tris pH 8.0, 200 mM NaCl, 0.1 mM phenylmethyl sulphonyl fluorideand 10 mM β-mercaptoethanol. The supernatant after centrifugation was loaded onto a Ni-NTA affinity column equilibrated with 20 mM Tris pH 8.0 and 200 mM NaCl. One millimolar TCEP was added immediately to the protein eluted using 300 mM imidazole. The eluted protein was reacted with EY-CBS according to the EY-CBS reaction protocol. The EY-CBS reacted protein was purified using a SP-Sepharose (GE Healthcare) cation exchange column equilibrated with a buffer containing 20 mM MES pH 5.5. The bound protein was eluted by linear gradient of 1 M NaCl. The fractions containing the target protein were pooled and cleaved overnight by thrombin to remove the purification tag. The cleaved protein was purified using a Supderdex 200 gel filtration column equilibrated with a buffer containing 20 mM Tris pH 8.0 and 200 mM NaCl.

### EY-CBS reaction

EY-CBS was synthesized as described by Zhang *et al*.^19^ The EY-CBS reaction, typically with 1 mg ml^−1^ fusion protein, was carried out at 23 °C for 1 h in 20 mM Tris HCl buffer at pH 8.0 and 200 mM NaCl. One millimolar TCEP was added to the reaction buffer to prevent oxidation of the cysteine during the reaction. Progress of the reaction was monitored by SDS–PAGE. The reaction was stopped and unreacted EY-CBS was removed by Superdex 200 gel filtration or SP-Sepharose ion exchange chromatography. The reacted and purified proteins were concentrated with an ultrafiltration kit and used for crystallization if necessary. The absorption spectrum of the protein after the EY-CBS reaction was measured with a JASCO UV-530 spectrophotometer.

### The PEG-Maleimide reaction

For the PEG-Maleimide reaction, 10 mM Maleimide-PEG_11_-Biotin (Thermo Scientific) or PEG-Maleimide 5000 (Nanocs) was added to fusion proteins that either had, or had not, been reacted with EY-CBS. The progress of the reaction was monitored by SDS–PAGE and the reaction was stopped by adding SDS–PAGE sample buffer containing 570 mM β-mercaptoethanol.

### Crystallization

Purified and concentrated proteins were used for crystallization. Initial crystallization conditions were screened using a crystallization robot (Mosquito, TTP Labtech). Unreacted 3,311 fusion protein was crystallized in a buffer containing 0.1 M Bis-Tris pH 6.5, 45% v/v PEG 400 and 10 mM hexamine cobalt (III) chloride. The crystals were frozen in liquid nitrogen. Addition of cryoprotectant was not necessary. After EY-CBS reaction and addition of excess amount of MBP, the 3,311-EY-CBS–MBP complex protein was purified by Superdex 200 gel filtration chromatography and crystallized in a buffer containing 0.1 M MOPS pH 6.5, 0.2 M magnesium chloride and 36% w/v PEG 2000. Crystals were frozen in a buffer containing the crystallization solution plus 14% PEG 2000 as the cryoprotectant. The 6,761 fusion protein was crystallized in a solution containing 10% w/v PEG 1000 and 10% w/v PEG 8000. Crystals were frozen in a buffer containing the crystallization solution plus 30% ethylene glycol as the cryoprotectant. The 8,155 protein reacted with EY-CBS was crystallized in a solution containing 1.84 M NaK phosphate pH 7.5. Crystals harvested with the crystallization buffer were flash frozen in a drop of paraffin oil (Hampton Research) using liquid nitrogen.

### X-ray diffraction data and structure refinement

Diffraction data were collected at Pohang Accelerator Laboratory, beam lines 5C and 7A. Diffraction images were integrated and scaled with HKL2000. All the structures were solved by the molecular replacement method using the PHASER software^43^. Initial phasing data were then refined using PHENIX^44^. The program COOT was used for manual correction of the structure. The crystallographic data are summarized in Table 2. The 3,311 crystals were merohedrally twinned with a twin fraction of 0.46. Initially we could not solve the structure by the molecular replacement technique because the diffraction data were incorrectly indexed for the P6_3_22 space group. Later we found that the crystals were almost perfectly twinned (Supplementary Fig. 21). Therefore, the data were re-indexed for space groups with lower symmetry. Promising solutions could be found for both the P6_3_ and P2_1_ space groups in molecular replacement calculations. However, refinement could not reduce the free R factor below 40% when the data were indexed for the P6_3_ space group. Therefore, they were finally indexed for the P2_1_ space group, and the structure was refined. The free R factor dropped from 0.369 to 0.261 after incorporation of the twin operator in the refinement protocol.

## Additional information

**Accession codes:** Atomic coordinates and diffraction data have been deposited in the Protein Data Bank with code numbers 5CBN, 5CBO, 5COC and 5EWX for the 3311+EY-CBS, 3311, 6761 and 8155+EY-CBS structures, respectively.

**How to cite this article:** Jeong, W. H. *et al*. Connecting two proteins using a fusion alpha helix stabilized by a chemical cross linker. *Nat. Commun.* 7:11031 doi: 10.1038/ncomms11031 (2016).

## Supplementary Material

Supplementary Information Supplementary Figures 1-21 and Supplementary References

## Figures and Tables

**Table 1:** Design of the fusion proteins.

Name	First protein	Second protein	Sequence of the fusion helix		
3,305	Protein A	mbp3–16	266*	QCAREAAREAAACD	13*
3,306	mbp3–16	Protein A	133	QCAREAAAREAACN	217
3,307	Protein A	mbp3–16	263	NCAQEAAAREAACD	13
3,308	mbp3–16	Protein A	129	ACILQAAAREAACN	217
3,309	Protein A	mbp3–16	266	QCAREAAARDLGCK	17
3,310	mbp3–16	Protein A	133	QCAREAAAREANCE	219
3,311	mbp3–16	Protein A	132	LCKNKAQQAAFYCI	227
3,312	mbp3–16	Protein A	125	NCRLAAILAKNKCQ	220
3,313	Protein A	mbp3–16	259	ACKLNAAQALGRCL	18
3,315	Protein A	mbp3–16	260	ACLNAAQAALGRCL	18
4,254	Protein A	mbp3–16	260	KCLNDALGAKLLCA	21
4,256	Protein A	mbp3–16	260	KCLNDAQAAKLLCA	21
4,257	Protein A	mbp3–16	259	ACKLNAAQALGRCL	18
4,258	Protein A	mbp3–16	259	ACKLNAAQAAGRCL	18
4,260	Protein A	mbp3–16	261	KCNDAAARALLECA	22
4,261	Protein A	mbp3–16	261	KCNDAALGAKLLCA	21
4,262	Protein A	mbp3–16	261	KCNDAAAGAKLLCA	21
6,758	Protein A	Calmodulin	260	KCLNDAEQIAEFCE	15
6,759	Protein A	Calmodulin	260	KCLNDEQIAEFKCA	16
6,760	Protein A	Calmodulin	259	ACKLNAQIAEFKCA	16
6,761	Protein A	Calmodulin	260	KCLNDAQAAAEECI	10

^*^Residue numbers are taken from the UniProt (protein A and calmodulin code number P38507 and P62158, respectively) and the GenBank (mbp3–16, AY326426) databases.

**Table 2:** Data collection and refinement statistics.

	3,311+ EY-CBS	3,311	6,761	8,155+ EY-CBS
*Data collection*				
Space group	P2_1_2_1_2_1_	P2_1_	I4_1_	C2
Resolution (Å)	2.30–50	2.80–50	2.70–50	2.60–50
				
Coordinations::				
a, b, c (Å)	64.3, 75.8, 111.4	85.4, 220.0, 85.4	98.8, 98.8, 31.7	89.5, 149.9, 55.8
α, β, γ (°)	90.0, 90.0, 90.0	90.0, 60.0, 90.0	90.0, 90.0, 90.0	90.0, 122.7, 90.0
R_sym_	0.063 (0.513)	0.051 (0.152)	0.114 (0.762)	0.079 (0.352)
*I/σI*	31.9	25.3	17.2	18.3
Completeness (%)	99.7 (99.2)	92.9 (80.0)	98.7 (97.0)	99.1 (98.5)
Redundancy	6.8	3.2	6.5	3.5
Search probes (PDB code)	1SVX, 1DEE	1SVX, 1DEE	2SPZ, 1CLL	1LYD, 1DEE
				
*Refinement*				
No. of reflections	24,741	62,287	4,460	35,397
R_work_/R_free_	0.194/0.262	0.207/0.250	0.206/0.259	0.200/0.259
Twin				
Operator	NA	h, -k, h-l	NA	NA
Fraction	NA	0.46	NA	NA
No. of protein molecules	1 (MBP)+1 (3311)	12	1	2
No. of atoms	4,330	16,233	978	3,451
Protein	4,188	16,116	966	3,408
Ligands	30	0	0	60
Water	112	117	12	43
Average B factor (Å^2^)	51.2	29.6	65.4	57.4
R.m.s. deviations				
Bond length (Å)	0.008	0.010	0.009	0.009
Bond angles (°)	1.080	1.081	1.105	1.451

EY-CBS, 3,3′-ethyne-1,2-diylbis-{6-[(chloroacetyl)amino]benzenesulfonic acid}; NA, not applicable; r.m.s., root mean square.

Highest resolution shell is shown in parenthesis.
