# Supplementary material for: Connecting two proteins using a fusion alpha helix stabilized by a chemical cross linker
Source: Nat Commun. 2016 Mar 16;7:11031. doi: 10.1038/ncomms11031 (PMC4799363; doi:10.1038/ncomms11031)
Supplement: Supplementary Information — Supplementary Figures 1-21 and Supplementary References [file ncomms11031-s1.pdf]

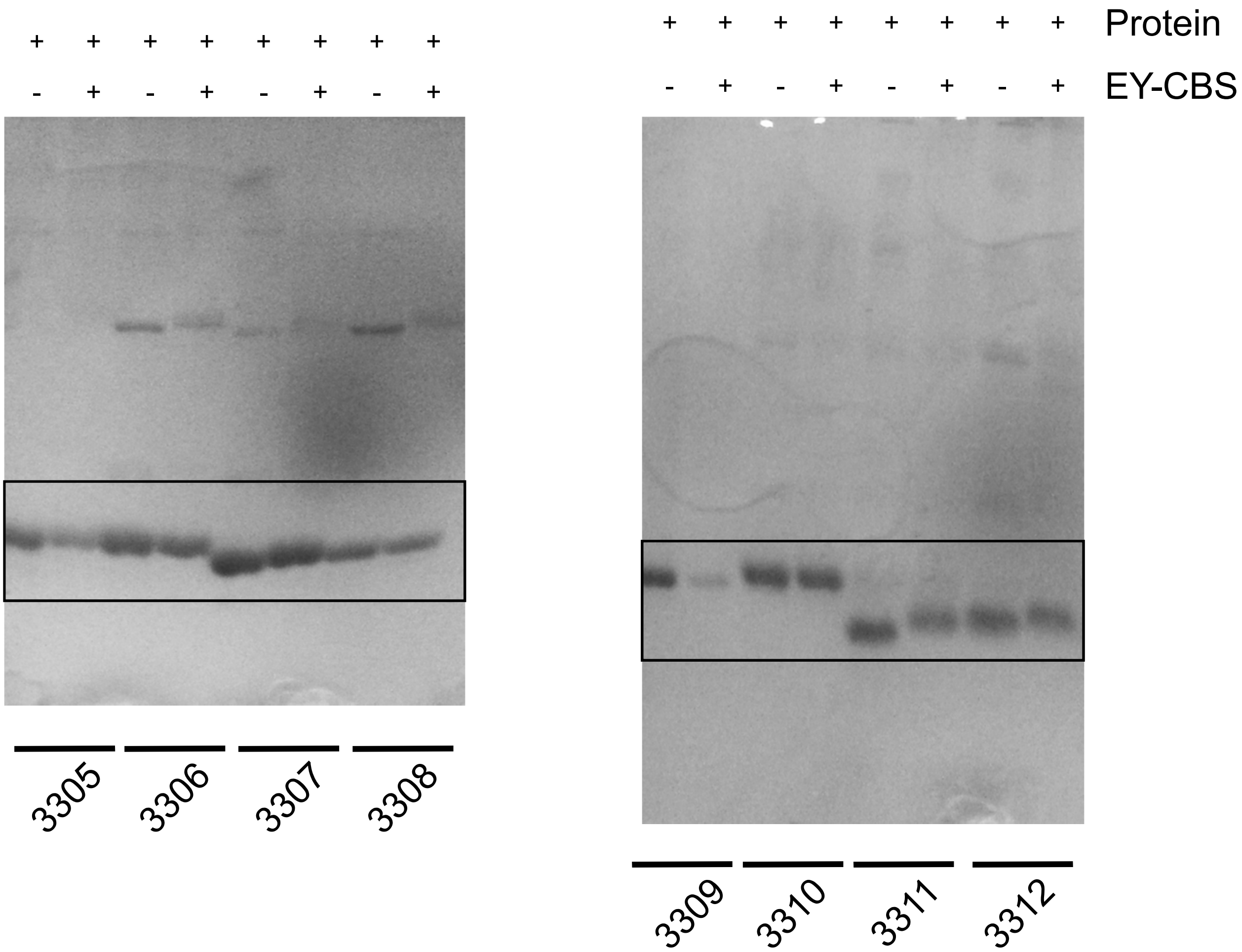

Supplementary Figure 1 | Full length SDS-PAGE for the designed fusion proteins with and without EY-CBS reaction. Uncropped SDS-PAGE comprising panels in Figure 2a.

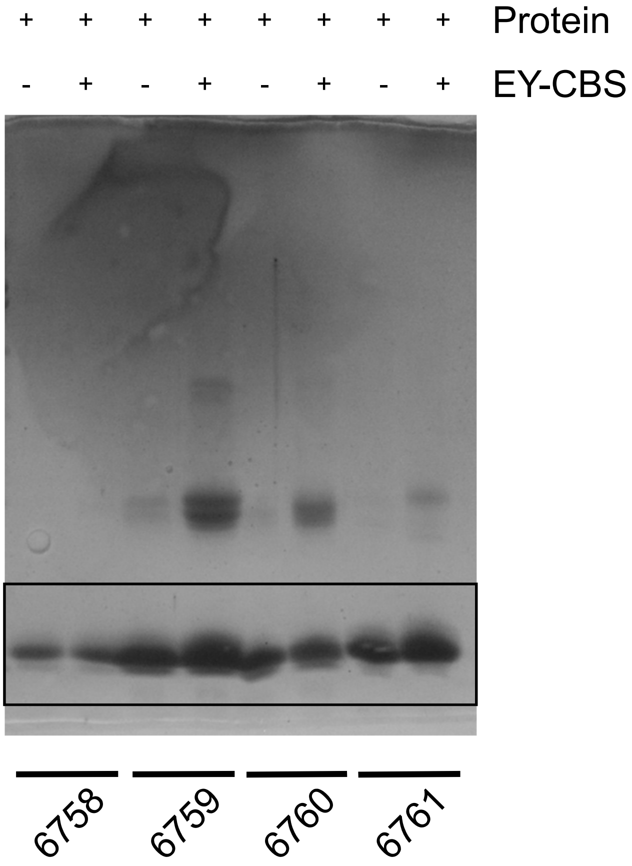

Supplementary Figure 2 | Full length SDS-PAGE for the designed fusion proteins with and without EY-CBS reaction. Uncropped SDS-PAGE comprising panels in Figure 2b.

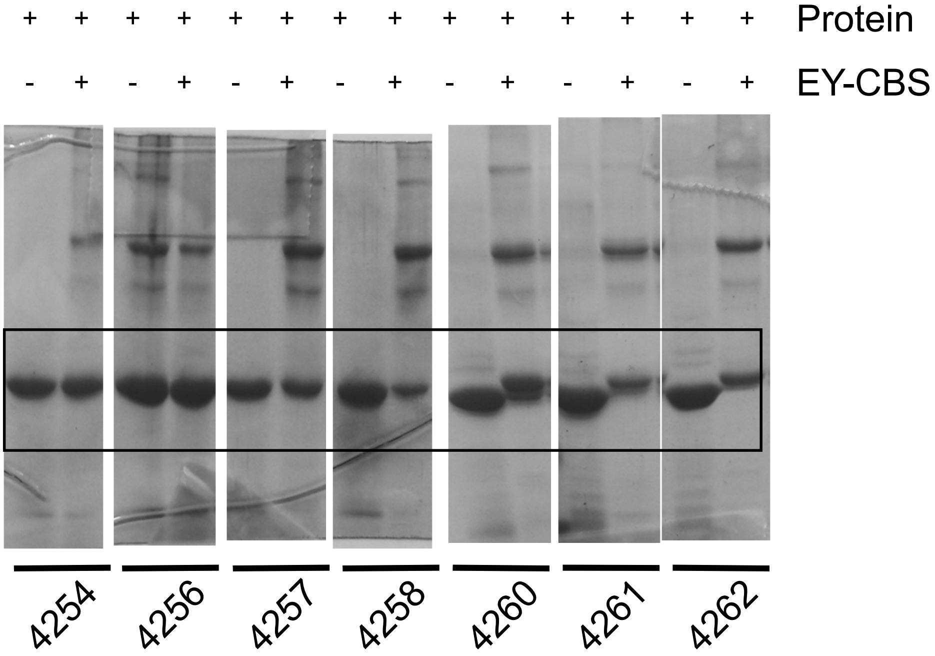

Supplementary Figure 3 | Full length SDS-PAGE for the designed fusion proteins with and without EY-CBS reaction. Uncropped SDS-PAGE comprising panels in Figure 2c.

mbp3-16

12 SDLGKKLLE
21 AAHAGQDDEVRILMANGADVNAMDNFGVTPLHL
54 AAYWGHFEIVEVLLKYGADVNASDATGDTPLHL
87 AAKWGYLGIVEVLLKYGADVNAQDKFGKTAFDI
120 SIDNGNEDLAEIL**cKN** 135
1218 **KaQQaAFYc**ILHLPNLNEEQRNGFIQSLKDDPS 1250
QSANLLAEAKKLNDAQAPK 1269

protein A

Supplementary Figure 4 | Amino acid sequence of the crystallized 3311 fusion protein. The amino acid sequence of the fusion helix is written in bold. Residues mutated for the EY-CBS reaction are written in lower case.

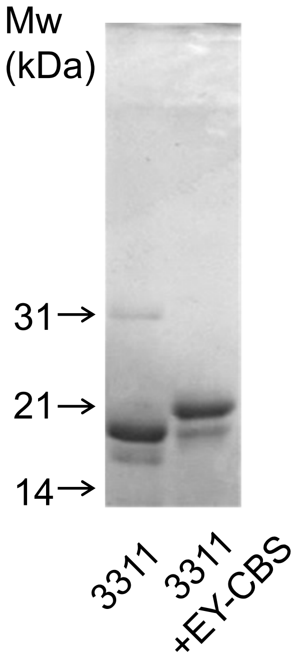

Supplementary Figure 5 | Full length SDS-PAGE for the 3311 fusion protein with and without EY-CBS reaction. Uncropped SDS-PAGE comprising panels in Figure 3a.

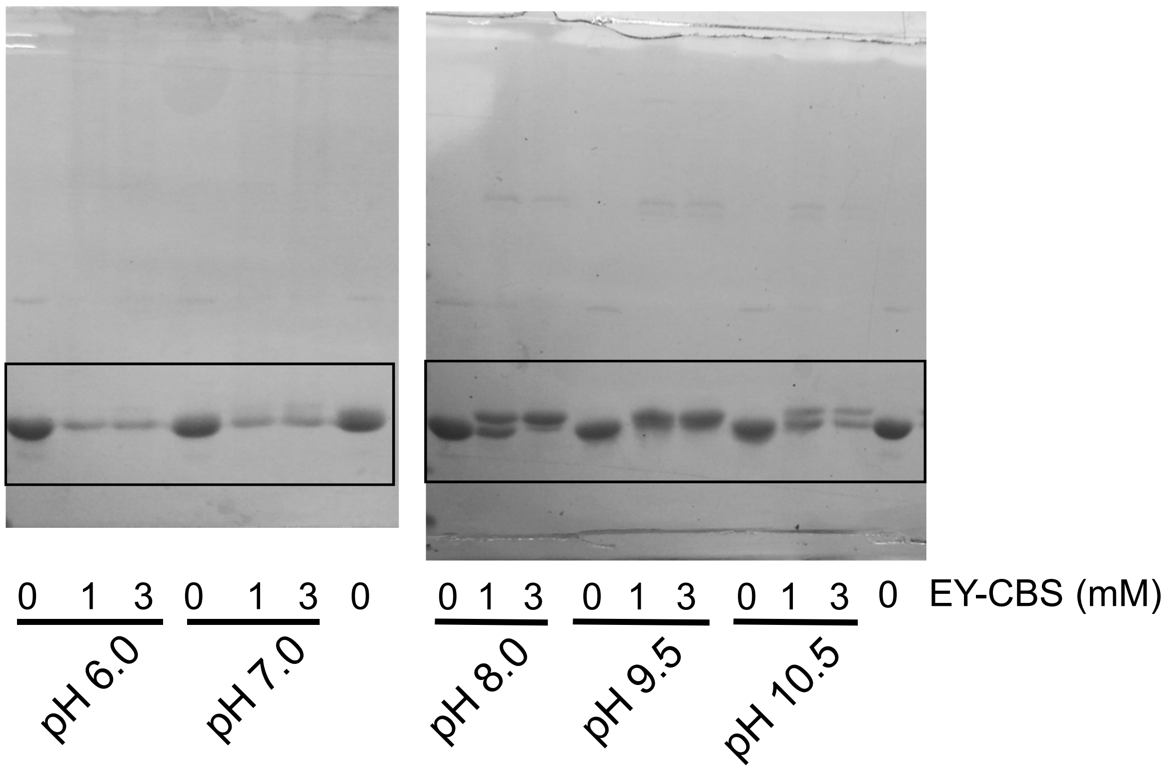

Supplementary Figure 6 | Full length SDS-PAGE for the 3311 fusion protein with and without EY-CBS reaction. Uncropped SDS-PAGE comprising panels in Figure 3c.

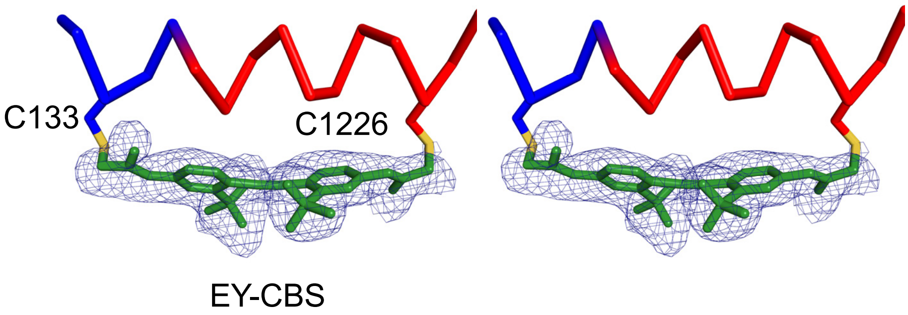

Supplementary Figure 7 | Stereoview of the simulated annealing omit map. The bound EY-CBS residue of the refined 3311-EY-CBS structure was omitted and the Fo-Fc map was calculated after simulated annealing. The map is contoured at the 2 s level.

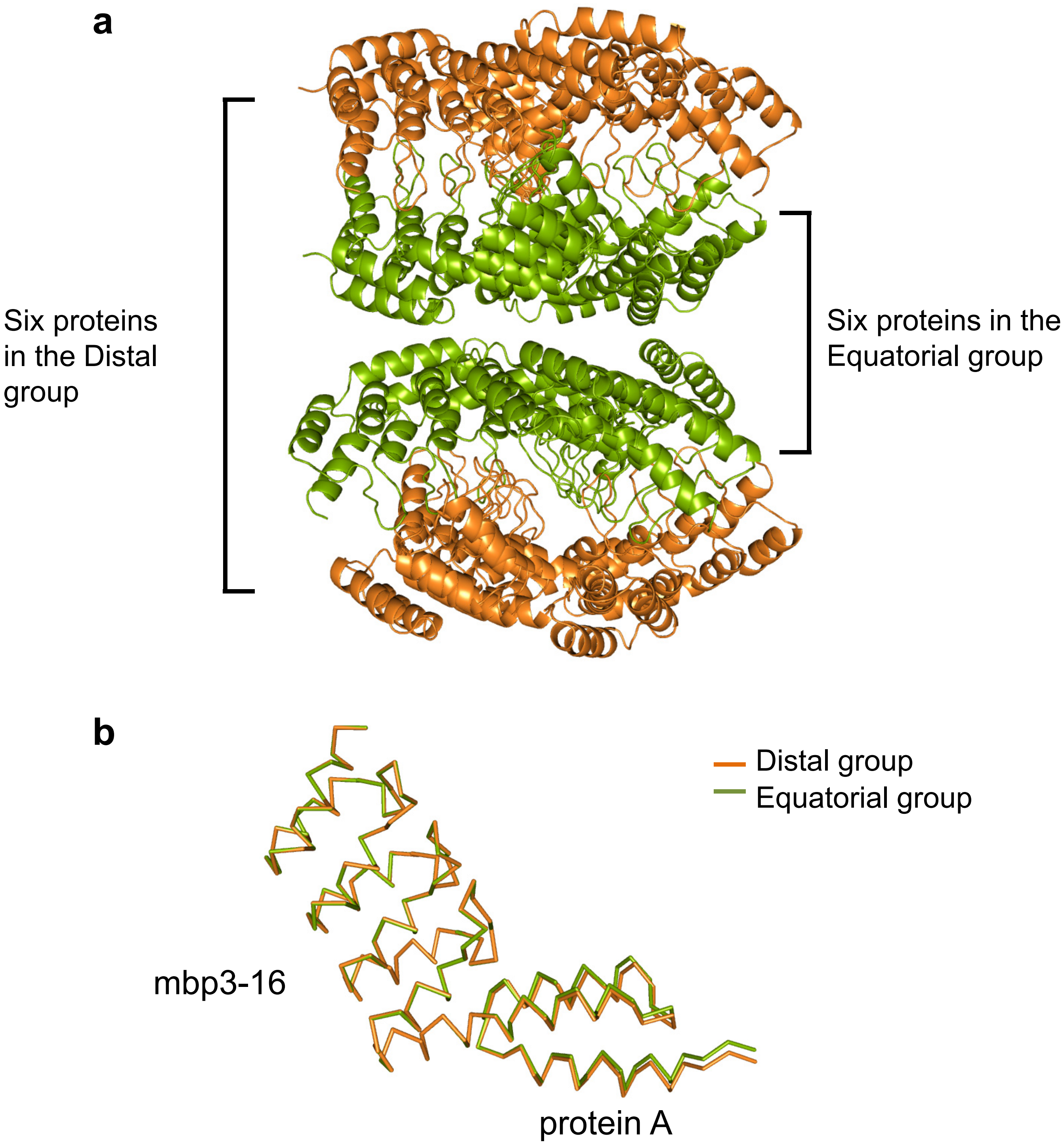

Supplementary Figure 8 | Arrangement of the proteins in the asymmetric unit of 3311 crystals. (**a**) The twelve proteins can be classified into two groups based on the crystal environment in which they are located. (**b**) Structural comparison of the "distal" and "equatorial" group proteins.

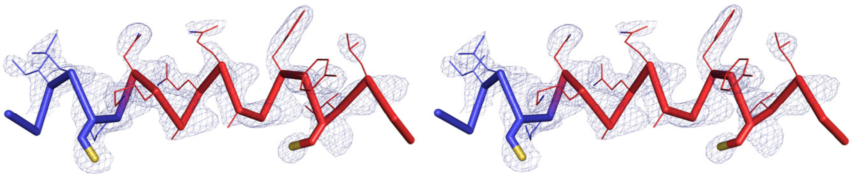

Supplementary Figure 9 | Stereo view of the simulated annealing omit map. The fusion helix of the refined 3311 structure was omitted and the Fo-Fc map was calculated after simulated annealing. The map is contoured at the 2 σ level.

protein A

222 VDNKFNKEQQNAFYEILHLPNLNEEQRNAFIQS

245 LKDDPSQSANLLAEAK**cLNDaQA** 267

1005 **aAEEc**IAEFKEAFSLFDKDGDGTITTKELGTVM 1037

RSLGQNPTEAELQDMINEVDADGNGTIDFPEFL 1070

TMMARKMK 1078

calmodulin

Supplementary Figure 10 | Amino acid sequence of the crystallized 6761 fusion protein. Residues of the fusion helix are written in bold. Those mutated for the EY-CBS reaction are written in lower case.

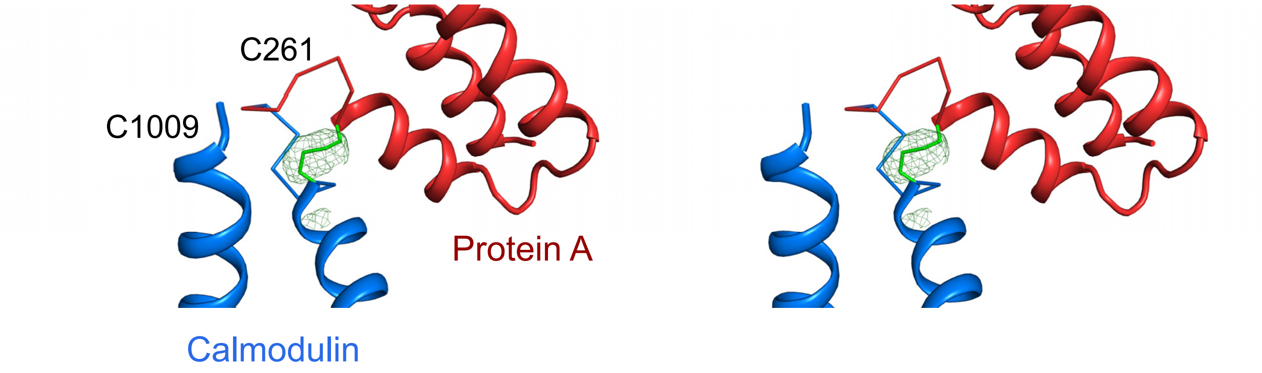

Supplementary Figure 11 | Stereoview of the simulated annealing omit map. Side chains of the Cys261 and Cys1009 residues of the refined 6761 structure were omitted, and the Fo-Fc map was calculated after simulated annealing. The map is contoured at the 2 σ level.

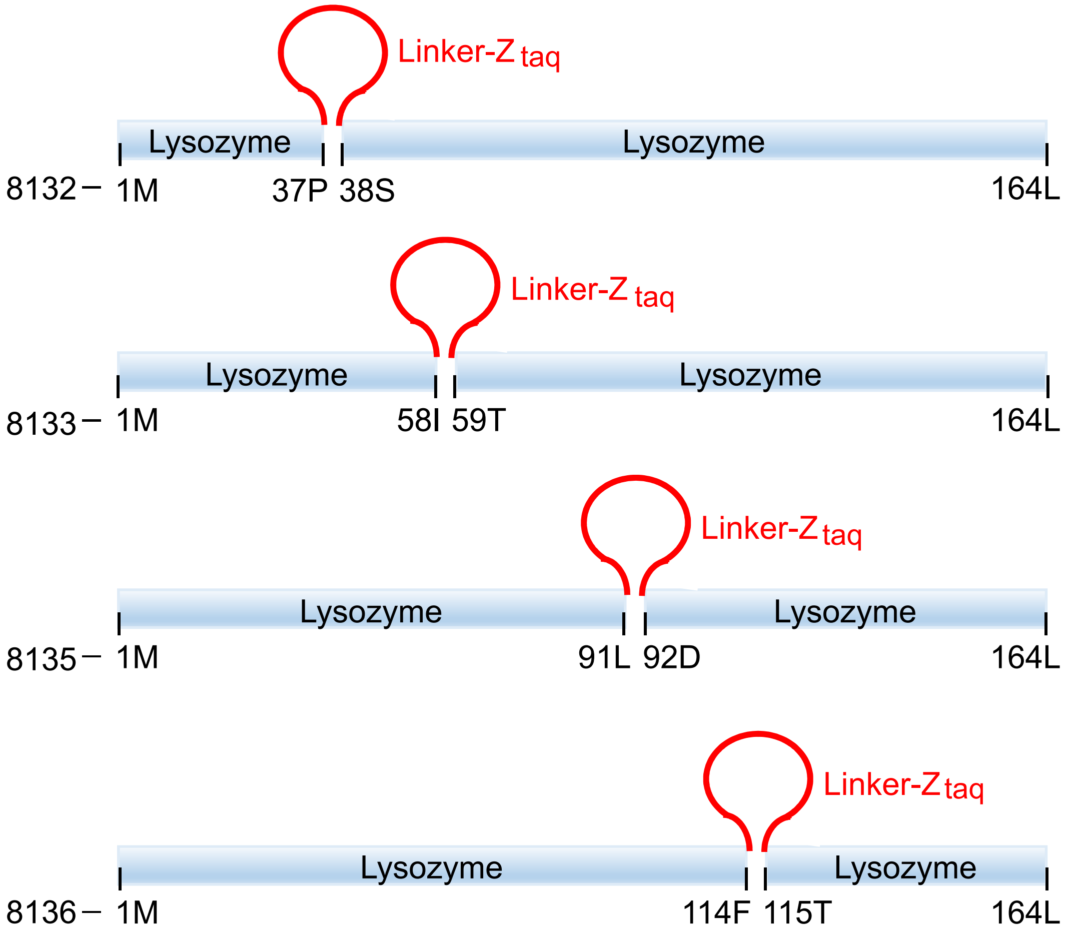

Supplementary Figure 12 | Schematic representation of T4 lysozyme-linker-Ztaq fusion proteins.

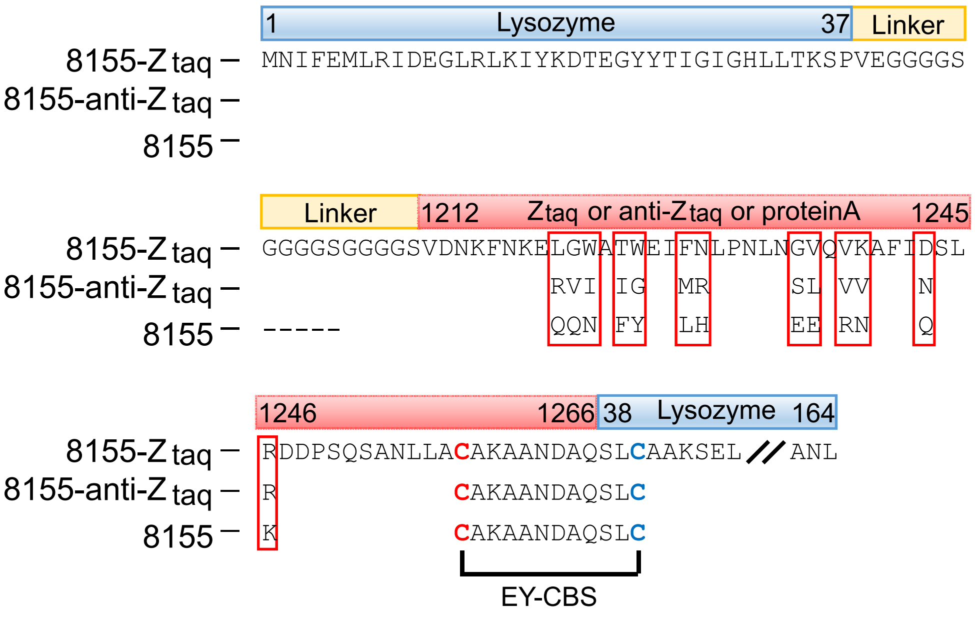

Supplementary Figure 13 | Amino acid sequences of the Lysozyme-protein A fusion proteins. Residue numbers of protein A are increased by 1000 for clarity.

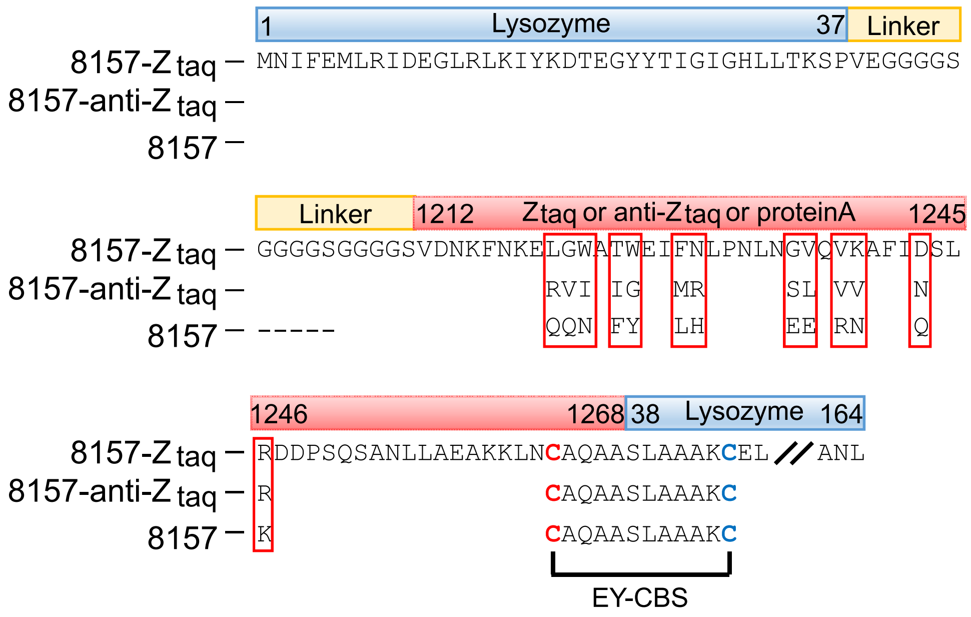

Supplementary Figure 14 | Amino acid sequences of the Lysozyme-protein A fusion proteins. Residue numbers of protein A are increased by 1000 for clarity.

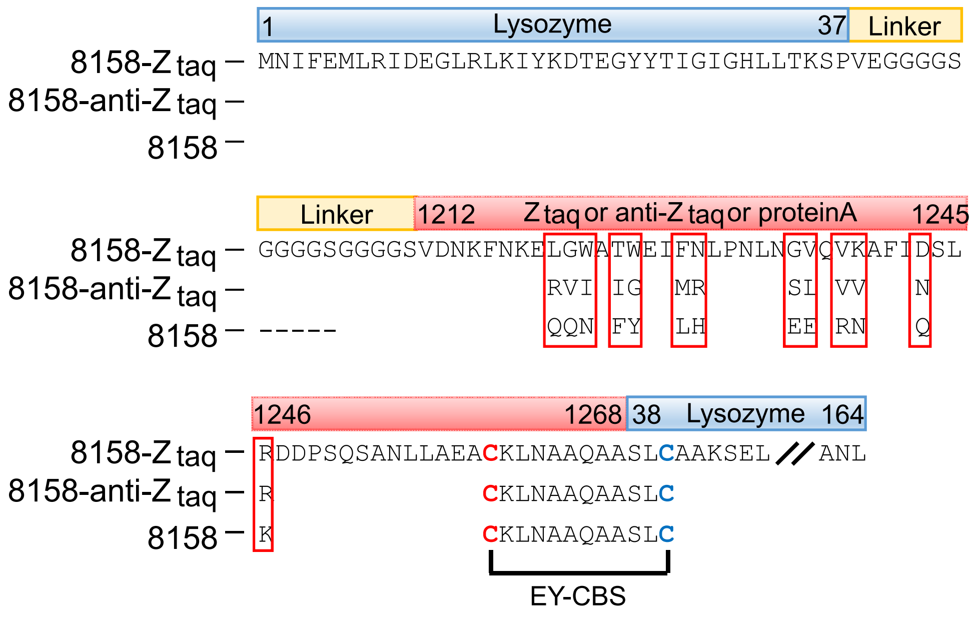

Supplementary Figure 15 | Amino acid sequences of the Lysozyme-protein A fusion proteins. Residue numbers of protein A are increased by 1000 for clarity.

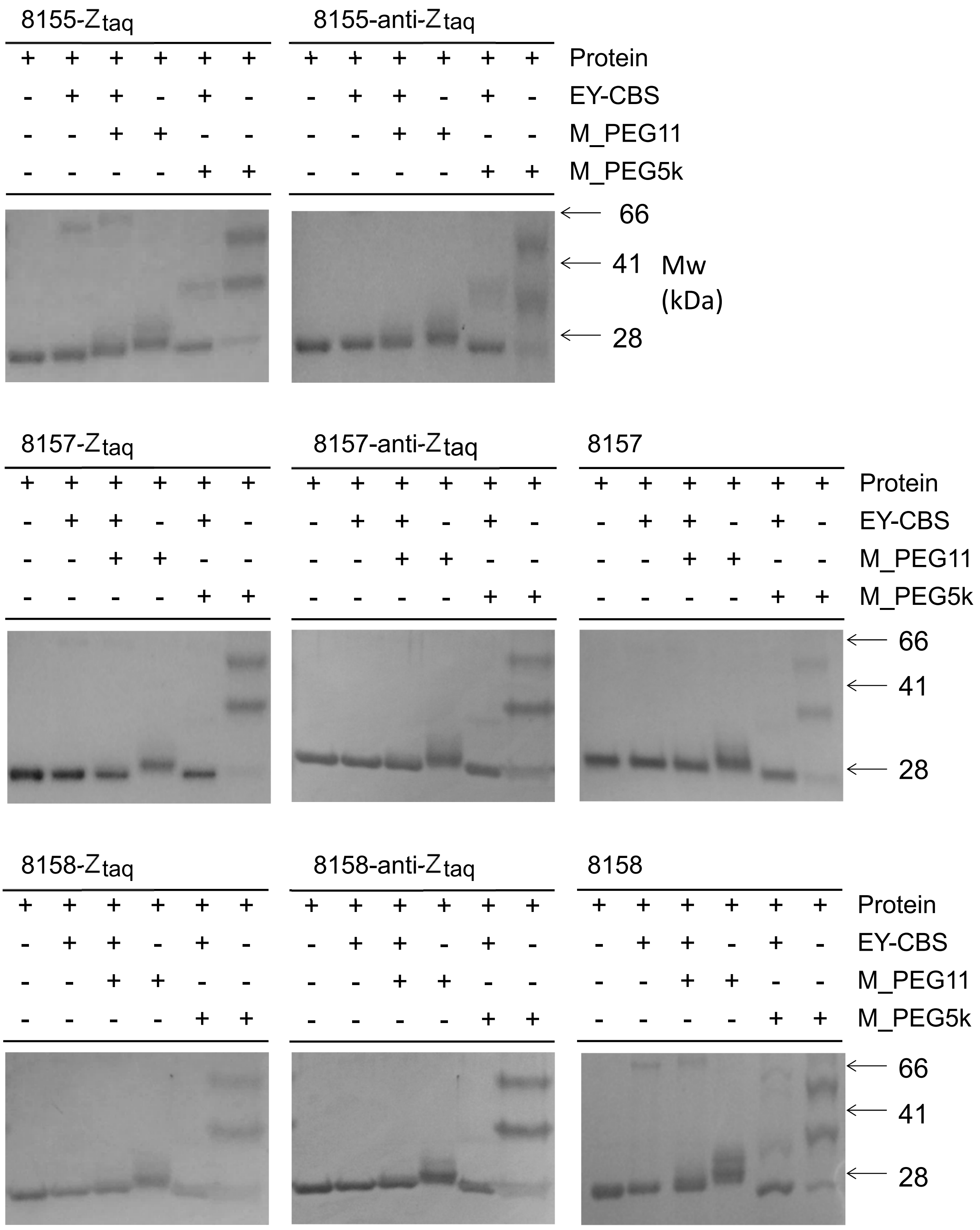

Supplementary Figure 16 | EY-CBS reactivity of the T4 lysozyme-protein A fusion proteins. M_PEG11 and M_PEG5k stand for Maleimide-PEG_{11}-Biotin and PEG-Maleimide 5000, respectively.

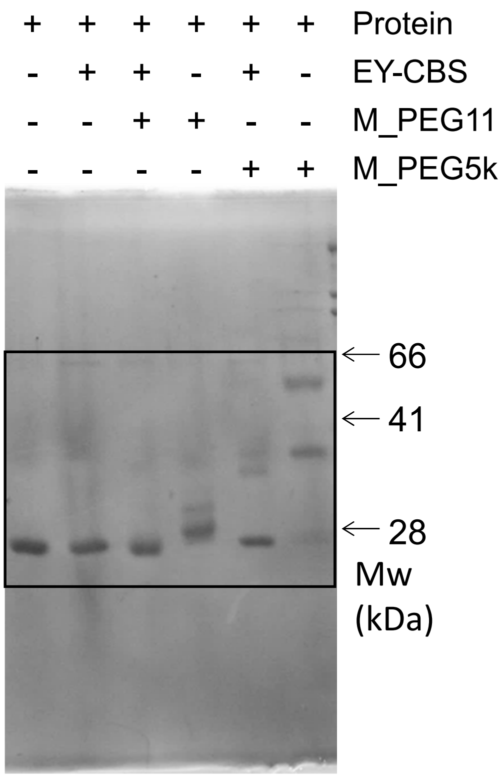

Supplementary Figure 17 | Full length SDS-PAGE for the 8155 fusion protein with EY-CBS reaction. Uncropped SDS-PAGE comprising panels in Figure 7b.

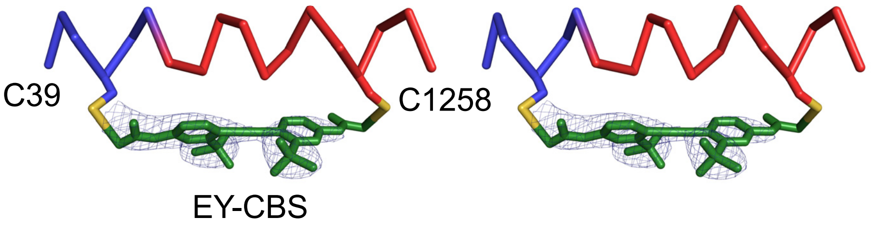

Supplementary Figure 18 | Stereoview of the simulated annealing omit map. The bound EY-CBS residue of the refined 8155-EY-CBS structure was omitted and the Fo-Fc map was calculated after simulated annealing. The map is contoured at the 2 σ level.

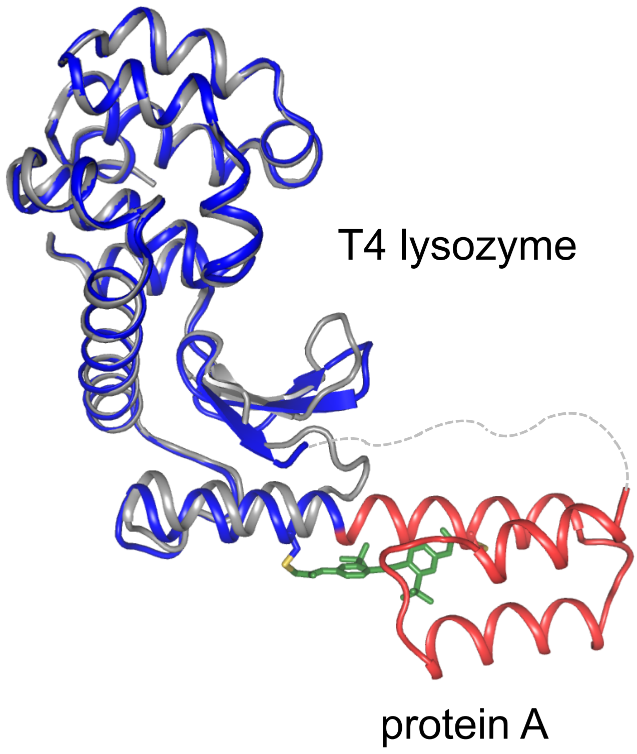

Supplementary Figure 19 | Comparison of the structures of T4 lysozyme and fusion protein 8155. The T4 lysozyme and protein A domains and the EY-CBS residue of the 8155 structure are colored in blue, red and green, respectively. Of 642 published structures of T4 lysozyme, the structure with PDB code number 1P7S gave the lowest Cα RMS difference with fusion protein 8155, and was drawn in grey [1].

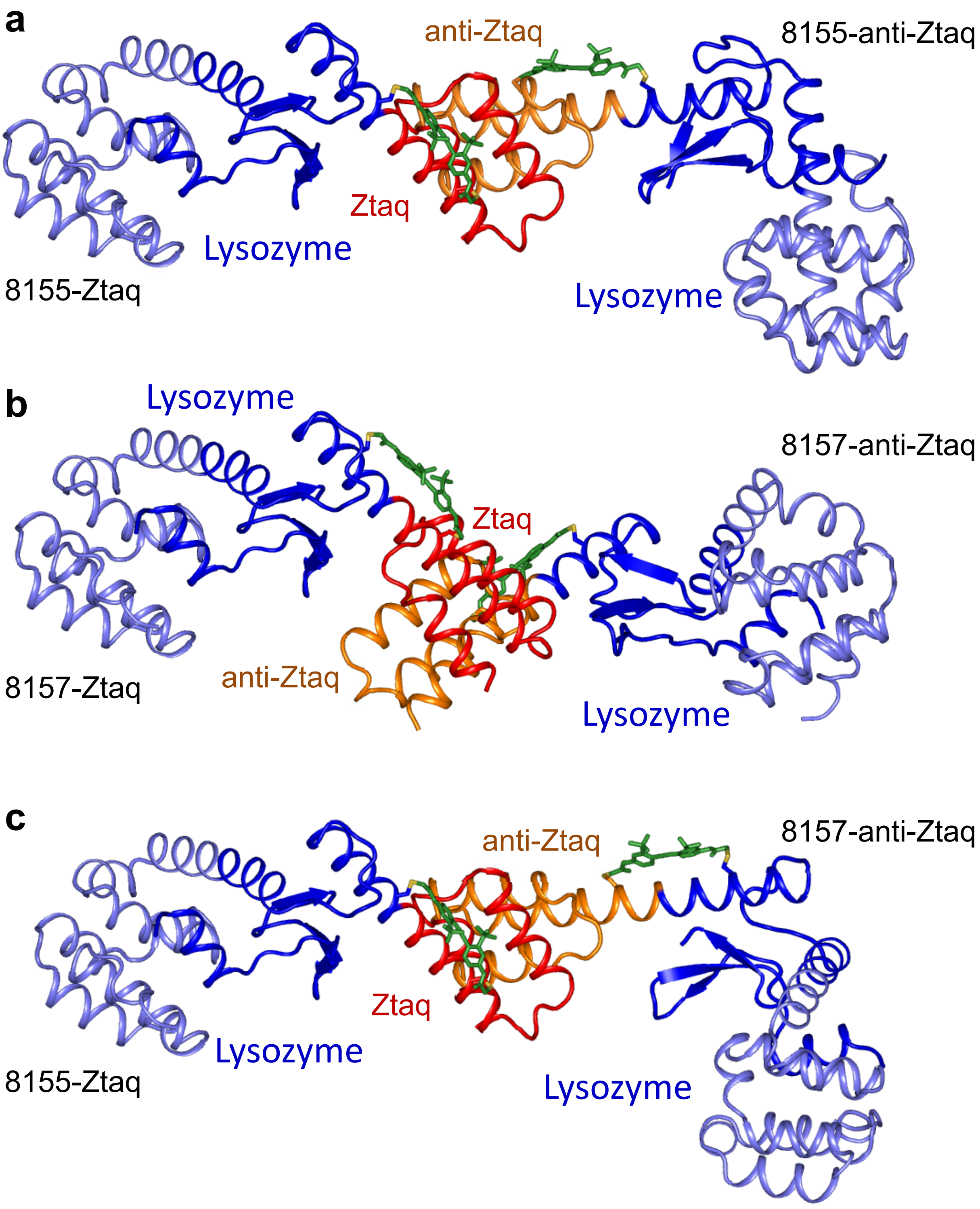

Supplementary Figure 20 | Models of the 8155-8155, 8157-8157 and 8155-8157 complexes mediated by Ztaq and anti-Ztaq heterodimerization. The N-terminal and C-terminal domains of T4 lysozymes are colored in blue and light-blue, respectively, and the Ztaq, anti-Ztaq domains and EY-CBS are drawn in red, orange and green, respectively. The structure of 8155-Ztaq is modelled using the crystal structure of 8155 and the published structure of Ztaq [2]. The model of 8157 is based on the crystal structure of 8155, extending the connecting helix by two amino acids. The previously published structure of the Ztaq-anti-Ztaq heterodimer was used for modeling the fusion heterodimers [2].

a

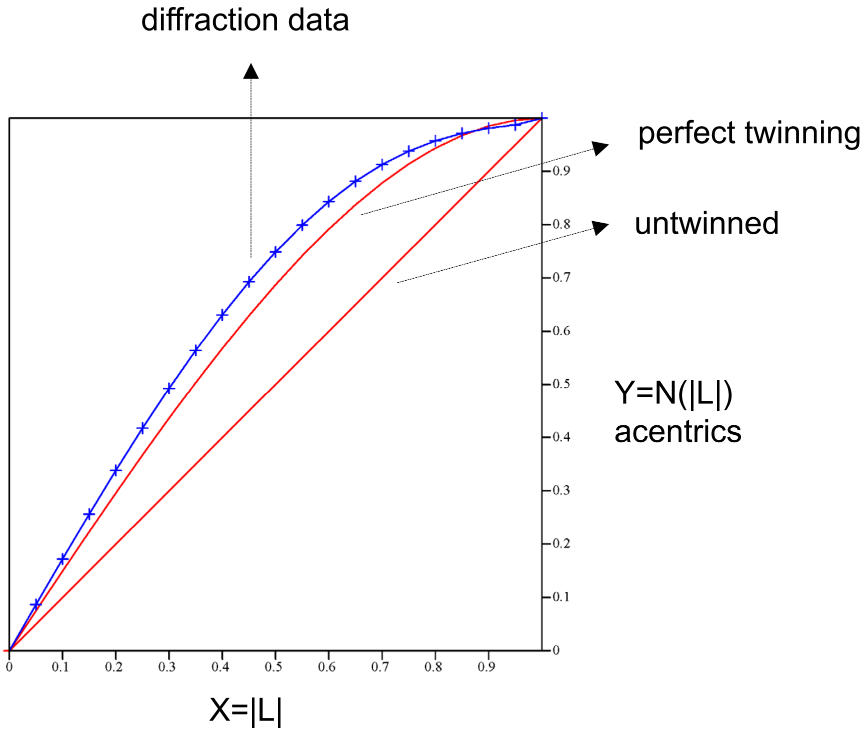

b

$\langle I^2\rangle/\langle I\rangle^2$	1.472 (untwinned: 2.0, perfect twin: 1.5)
$\langle F\rangle^2/\langle F^2\rangle$	0.900 (untwinned: 0.785, perfect twin: 0.885)
$\langle \lvert E^2-1\rvert \rangle$	0.507 (untwinned: 0.736, perfect twin: 0.541)
$\langle \lvert L\rvert \rangle$, $\langle L^2\rangle$	0.331, 0.160
Multivariate Z score L-test: 19.890	
Britton alpha	0.433
H alpha	0.436
ML alpha	0.435

Supplementary Figure 21 | Twinning analysis of the diffraction data for 3311 crystals. (**a**) DATAMAN Local Intensity Statistics plots. The data were analyzed on the UCLA-DOE LAB server (http://services.mbi.ucla.edu/Twinning) [3]. (**b**) The twin analysis parameters of the 3311 data calculated by the Xtriage program implemented in the PHENIX software package [4].

Supplementary References

1 Mooers, B. H. *et al.* Repacking the Core of T4 lysozyme by automated design. *J. Mol. Biol.* **332**, 741-756 (2003).

2 Lendel, C., Dogan, J. & Hard, T. Structural basis for molecular recognition in an affibody:affibody complex. *J. Mol. Biol.* **359**, 1293-1304 (2006).

3 Padilla, J. E. & Yeates, T. O. A statistic for local intensity differences: robustness to anisotropy and pseudo-centering and utility for detecting twinning. *Acta Crystallogr. D Biol. Crystallogr.* **59**, 1124-1130 (2003).

4 Adams, P. D. *et al.* PHENIX: a comprehensive Python-based system for macromolecular structure solution. *Acta Crystallogr. D Biol. Crystallogr.* **66**, 213-221 (2010).
